# An improved deep learning model for predicting daily PM2.5 concentration

**DOI:** 10.1038/s41598-020-77757-w

**Published:** 2020-12-02

**Authors:** Fei Xiao, Mei Yang, Hong Fan, Guanghui Fan, Mohammed A. A. Al-qaness

**Affiliations:** 1 School of Public Health, Southern Medical University, Guangzhou, 510515 Guangdong China; 2 State Key Laboratory of Information Engineering in Surveying, Mapping and Remote Sensing, Wuhan University, 129 Luoyu Road, Wuhan, 430079 China; 3 General Hospital of Centeral Theater Command of PLA, 627 Luoyu Road, Wuhan, 430079 China

**Keywords:** Ecology, Environmental sciences

## Abstract

Over the past few decades, air pollution has caused serious damage to public health. Therefore, making accurate predictions of PM2.5 is a crucial task. Due to the transportation of air pollutants among areas, the PM2.5 concentration is strongly spatiotemporal correlated. However, the distribution of air pollution monitoring sites is not even making the spatiotemporal correlation between the central site and surrounding sites vary with different density of sites, and this was neglected by previous methods. To this end, this study proposes a weighted long short-term memory neural network extended model (WLSTME), which addressed the issue that how to consider the effect of the density of sites and wind conditions on the spatiotemporal correlation of air pollution concentration. First, a number of nearest surrounding sites were chosen as the neighbor sites to the central site, and their distance, as well as their air pollution concentration and wind condition, were input to multilayer perception (MLP) to generate weighted historical PM2.5 time series data. Second, historical PM2.5 concentration of the central site and weighted PM2.5 series data of neighbor sites were input into a long short-term memory (LSTM) to address spatiotemporal dependency simultaneously and extract spatiotemporal features. Finally, another MLP was utilized to integrate spatiotemporal features extracted above with the meteorological data of the central site to generate the forecasts future PM2.5 concentration of the central site. Daily PM2.5 concentration and meteorological data on Beijing–Tianjin–Hebei from 2015 to 2017 were collected to train models and to evaluate its performance. Experimental results with three existing methods showed that the proposed WLSTME model has the lowest RMSE (40.67) and MAE (26.10) and the highest p (0.59). Further experiments showed that in all seasons and regions, WLSTME performed the best. This finding confirms that WLSTME can significantly improve PM2.5 prediction accuracy.

## Introduction

Over the past few decades, rapid economic growth worldwide has caused severe air pollution, which has elicited extensive global attention. PM2.5 (particulate matter with a diameter less than 2.5 um), as an important component of air pollutants, is related to cardiopulmonary and other systemic diseases because it penetrates the respiratory system^1,2^. According to a recent World Health Organization (WHO) study^3^, approximately 90% of people breathe air that does not comply with WHO Air Quality Guidelines, and about 3 million deaths worldwide are caused by outdoor air pollution in 2012.

Considering the proven negative effect of air pollution, forecasting daily PM2.5 concentration must be provided to control air pollution and combat health problems. Many studies have established unique approaches for PM2.5 prediction. These methods can be divided into physical and empirical models. Physical models, such as CMAQ^4^ and WRF/Chem-MADRID^5^, can provide explicit insights into the physical–chemical processes of the diffusion and transformation of multiple pollutants and present the direct linkage between pollutant emission and air pollution. However, these chemical transport models are dependent on a priori knowledge, which may cause errors.

Empirical models demonstrate the relationships between dependent and multiple independent variables based on historical data. Empirical methods mainly include regression models and machine learning algorithms. Classical regression models, such as multiple linear regression^6^, land use regression^7^, and autoregressive moving average^8^, are relatively simple. These models can just fit linear relationships between inputs and outputs, which are determined by their structures.

By contrast, many machine learning models have been applied widely for their great ability to handle nonlinear relationships, and they generally provide satisfactory performance. Representative models include artificial neural network^9,10^, recursive neural network^11^, support vector regression^12^, and hybrid model^13,14^. Recently, long short-term memory neural network (LSTM)^15^ has been used extensively for processing time-series data due to its capability of simulating long and short-term tendencies simultaneously. Li et al.^16^ constructed a method of using the historical concentrations of all sites as the inputs of the LSTM layer and integrating auxiliary variables by a fully-connected layer. Qin et al.^17^ proposed a combined model for forecasting a city’s PM2.5 concentration. The model used a convolutional neural network to extract features of input data of all sites automatically, and LSTM to consider the time dependency. Apart from those studies considering all stations, many studies addressed the spatial correlation by considering the most related sites. K Nearest Neighbors method was extensively employed to determine the K nearest sites to the target site^18–20^, and their meteorological conditions and air quality data were input to neural networks to capture spatial correlation. However, the distribution of monitoring stations is extremely uneven, causing the density of sites is quite different for different areas. The lower the station density, the farther the geographical distance of the selected K nearest sites to the target site, therefore the lower their effect degree. However, most of the existing studies neglected the importance of station density. Considering that air pollutants are transported based on wind, some studies further combined the geographical distance with wind conditions and generated weights of each surrounding site to represent their affect degree to the target site. For example, a space–time support vector regression model (STSVR) was proposed in Yang et al.^21^, which constructed Gauss vector weight function to combine the distance of surrounding sites and wind direction effects. Through integrating the weights and PM2.5 concentration of the surrounding sites, the spatial dependence was introduced into the SVR model. Similarly, through a linear combination between the wind direction and geographical distance, Li et al.^22^ proposed a wind field distance definition to represent the spatial correlation degree between two sites. Furthermore, Li et al.^23^ introduced the wind speed into the definition of wind field distance. Both of these studies enhanced the interpolation accuracy and indicated that the combination of wind and geographical distance has great potential for determining spatial dependence.

To deeply simulate the wind and geographical distance impacts on spatial correlation, the current study proposed a weighted LSTM neural network extended model (WLSTME) to forecast daily PM2.5 concentration. First, the K nearest stations were chosen as K neighbors of the target site, and local MLP was utilized to calculate the weighted PM2.5 concentration time series based on the historical PM2.5 concentration observation, geographical distance, and historical wind condition of the K neighbors. Second, the weighted PM2.5 concentration series data of the selected neighbor stations and the historical PM2.5 observation of the target station were fed into LSTM layers, which simultaneously simulated spatial and temporal dependencies and extracted spatiotemporal features. Finally, another MLP network was used to integrate the obtained spatiotemporal features with auxiliary variables, including meteorological variables and time stamp data, and generate the prediction of the next day’s PM2.5 concentration of the target station. Through the nonlinear combination by MLP, the proposed model can more effectively simulate the impact of station density and wind on spatial dependency, and avoid the remote sites’ interference to the prediction accuracy. Daily average PM2.5 concentration and meteorological data of Beijing, Tianjin, and Hebei in China collected from 2015 to 2017 were employed as experimental data here. We conducted comparison experiments with the other three methods, and the results demonstrated the effectiveness of our model in predicting daily PM2.5 concentration.

The main contribution of this study :

1. This study innovatively uses the neural network MLP to combine the wind, distance, and PM2.5 concentration. Comparative experiments indicated that the MLP network outperforms the Gaussian form; therefore, the proposed WLSTME model can more effectively simulate the impact of station density and wind on spatial dependency, and avoid the interference of the remote sites.
2. Spatial and temporal correlations are complex and comprehensive. For example, the impact of adjacent sites affects not only the previous day, but also the past few days, and the impact may change over time. In our model, the historical data of the central station and adjacent sites are integrated and input into LSTM. From the results, the proposed method is found more effective in extracting spatiotemporal features and performs with higher daily PM2.5 prediction accuracy than other methods.
3. Since the future weather condition is also highly related to the future PM2.5 concentration, and by integrating the weather forecast data, the MLP weather forecast optimizer can help the WLSTME to further reached high forecast accuracy of PM2.5.

The rest of this paper is organized as follows. Section “Materials and methods” introduces the data and methods. Section “Results and discussion” presents the experimental results and related discussions. Section “Conclusion” provides the conclusion.

## Materials and methods

### Study area

**Figure 1:**
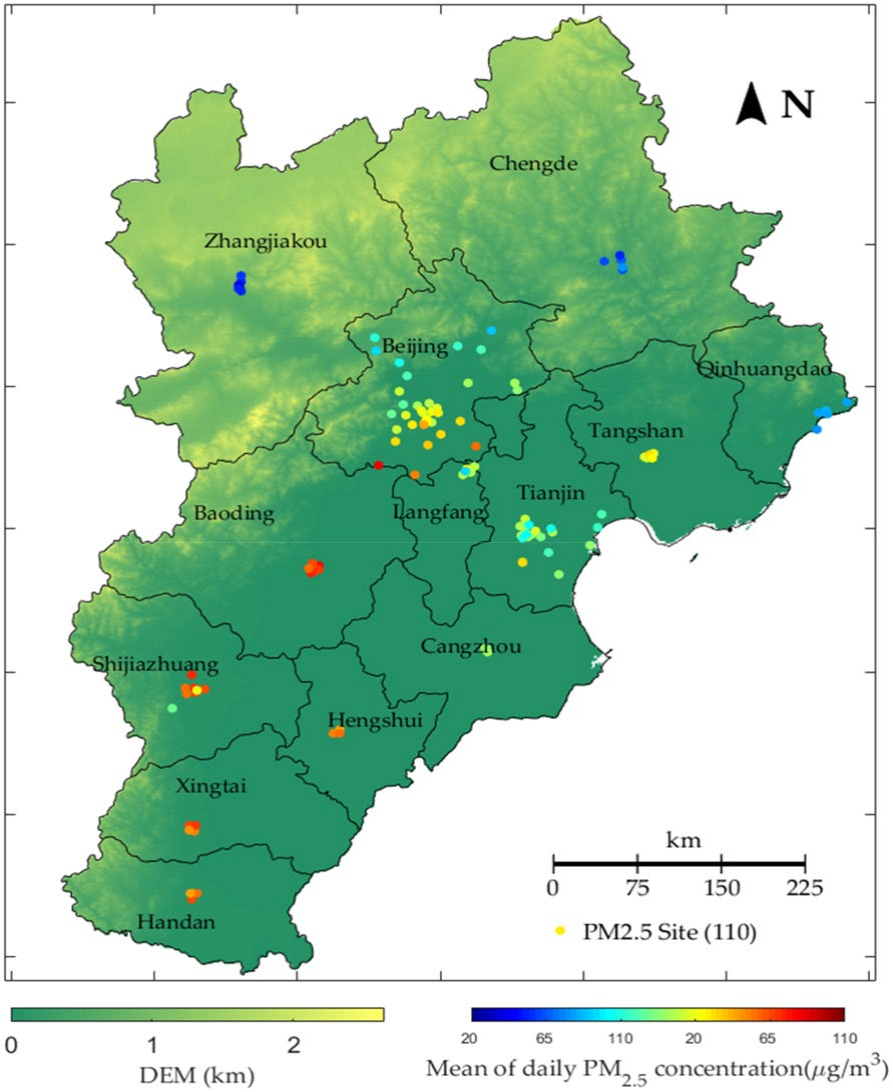
Locations of air quality stations in BTH region. The color represents the rank of the average daily PM2.5 concentration during 01 Jan., 2015 to 31 Dec., 2017 as described in the bottom of the Figure. (This Figure is drawn by using Matlab software).

The Beijing–Tianjin–Hebei (BTH) region of China is one of the most economical and active areas in China, containing Beijing, Tianjin, and 11 cities of Hebei Province. According to CSY (2018), the regional GDP of BTH in 2017 contributed 9.77% to the total GDP of China, and its population accounted for 8.09%. However, serious air pollution occurred, and its damage to public health cannot be ignored. According to the Ministry of Environmental Protection (MEP) (2018), among the top 20 most polluted cities, 9 cities belonged to Hebei Province, and Tianjin and Beijing ranked 15th and 19th, respectively. Thus, this study adopted the BTH region as the study area for constructing the PM2.5 concentration forecasting model. Figure 1 shows the Locations of air quality stations in the BTH region.

### Data

The research data and their description used in this study are listed in Table 1. We collected data from the period of 01 Jan. 2015 to 31 Dec. 2017. The variables included the PM2.5 concentration, meteorological data, the latitude and longitude of the monitoring station, and time stamp data, i.e., the month and week of the observation.

**Table 1:** List of the research data.

Data Type	Parameter	Unit	Frequency	Spatial Resolution	Source
Ground measured	PM2.5	$\mathrm{\mu g}/{m}^{3}$	hour	station	BMEMC (http://www.bjmemc.com.cn/) and http://pm25.in/
	Latitude and longitude of the monitoring station	°	/	/	
	month of the observation	/	/	/	
	week of the observation	/	/	/	
Near-real time analysis	U component of wind^1^	m/s	6 h	~ 10 km	European Centre for Medium-Range Weather Forecasts (ECMWF) (http://apps.ecmwf.int/datasets/data/cams-nrealtime/levtype=sfc/)
	V component of wind^2^	m/s	6 h	~ 10 km	
	Mean sea level pressure	Pa	6 h	~ 10 km	
	Dew point temperature	$\mathrm{^\circ{\rm C} }$	6 h	~ 10 km	
	Total column water vapor	$\mathrm{kg}/{m}^{2}$	6 h	~ 10 km	
	Temperature	$\mathrm{^\circ{\rm C} }$	6 h	~ 10 km	
Satellite products	MOD11A1 temperature data	$\mathrm{^\circ{\rm C} }$	1 day	1 km	https://modis.gsfc.nasa.gov/

^1^Zonal wind speed.

^2^Meridional wind speed.

1. *PM2.5 concentration data*: There are 110 air pollution monitoring stations distributed in the BTH region, as shown in Fig. 1. The hourly concentration of PM2.5, PM10, CO, NO2, O3, and SO2 are recorded and published by the Beijing Municipal Environmental Monitoring Center and http://pm25.in/. Station parameters are also recorded, including the latitude and longitude of the station, the month and the week of the observation. Here, we used the mean of the PM2.5 concentration from 0:00 to 23:00 to represent the daily PM2.5 concentration, and the rank of the average daily PM2.5 concentration from 2015 to 2017 is indicated by the color in Fig. 1. It can be found that air pollution is more serious in the south area.
2. *Meteorological data*: The meteorological variables considered in this study included temperature, wind speed, wind direction, mean sea level pressure, dew point temperature and total column water vapor. Meteorological data were obtained from CAMS Near-real-time dataset of ECMWF with around 10 km spatial resolution. Particularly, this dataset only provides $U$ and *V* component of wind (i.e., zonal and meridional wind speed), thus, the wind speed and wind direction were obtained by the Eqs. (1) and (2).
  1 $$wind \; speed=\sqrt{{u}^{2}+{v}^{2}}$$
  2 $$wind \; direction=\frac{\pi }{2}-{\mathit{tan}}^{-1}\frac{v}{u}$$

where $u$ and *v* refer to $U$ and $V$ components of wind, respectively. In addition, to further improve the spatial resolution of temperature, the 1 km spatial resolution Modis temperature product (MOD11A1) were also collected.

#### Data pre-processing

Due to critical failure or temporary power cutoff, missing values for a long or short periods happened in air pollution monitoring stations^24^. Similarly, some missing values occur in the ECMWF dataset. We got rid of data of 20 stations which have over 10% missing values in PM2.5 concentration data or ECMWF data. The missing PM2.5 concentration values of remaining stations were interpolated by inverse distance weight method.

Next, time stamp data, including month and day, were one-hot encoded; PM2.5 concentration and meteorological data were centralized and standardized in accordance with Eq. (3):

3 $${x}_{i}^{*}=\frac{{x}_{i}-\stackrel{-}{x}}{\sigma }$$

where ${x}_{i}$ and ${x}_{i}^{*}$ represent the original and transformed observation of a factor $x$*,* respectively; $\stackrel{-}{x}$ and $\upsigma$ are the mean and standard deviation of all observations, respectively.

Finally, the temperature data collected from MOD11A1 and ECMWF dataset were merged together to enhance its reliability. Since the spatial resolution of MOD11A1 data is higher, we used MOD11A1 data as basis, and filled its missing values by ECMWF data. Linear regression model was built between the centralized and standardized ECMWF temperature data ($cs\_E$) and MOD11A1 data ($cs\_M$) as the Eq. (4) shows,

4 $$c{s}_{M}=0.953842*c{s}_{E}-0.074635$$

The ${R}^{2}$ value of this model was 0.91, indicating a great consistency between them. Therefore, we filled the missing values of $cs\_M$ by the regression results of corresponding $cs\_E$ values.

### Methods

**Figure 2:**
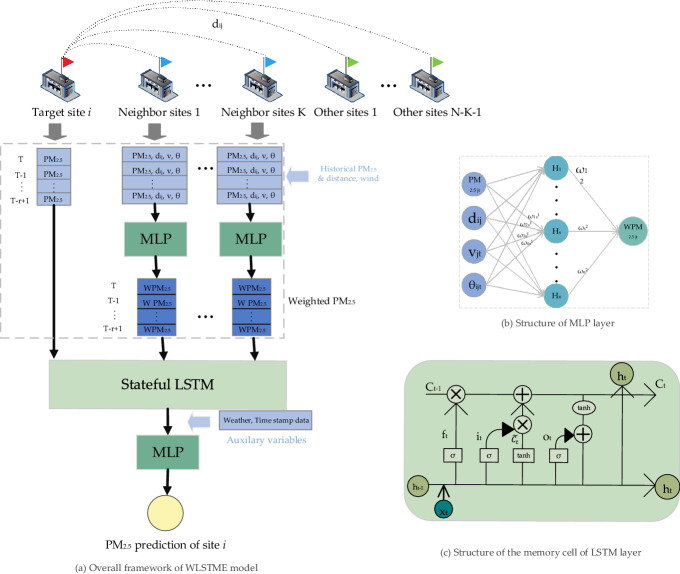
(**a**) Overall framework of WLSTME. The red, blue and green flags stand for the central site,* K* nearest sites, and other sites, respectively. *r* represents the time lag; (**b**) Structure of MLP layer; (**c**) Structure of the memory cell of LSTM layer. *x*_*t*_ and *h*_*t*_ are the inputs.

Figure 2 shows the overall framework of the proposed WLSTME model. As shown in Fig. 2, the model is a hybrid model that integrates three neural networks, including:

1. An MLP network to generate the weighted PM2.5 by combing wind speed and direction, geographical distance with historical PM2.5 concentration.
2. An LSTM network to address spatiotemporal dependency simultaneously and extract spatiotemporal features.
3. Another MLP network to optimize the prediction by integrating the spatiotemporal features and weather forecast data.

In detail, the three network works together to form an organic whole to achieve the daily PM2.5 prediction. Firstly, the MLP was used to combine historical wind speed and wind direction, the geographical distance between central sites and neighbor sites with corresponding days’ PM2.5 data of neighbor sites, and generate the weighted PM2.5. Then, the generated weighted PM2.5 of the neighbor sites in the past ten days were merged with the historical PM2.5 data of the central station, and input into the LSTM network to address the spatial and temporal dependence simultaneously and extract spatiotemporal features. Finally, another MLP was used to conduct bias adjustment by integrating the spatiotemporal features produced by LSTM with the central site’s meteorological data and time stamp data.

The input of WLSTME model consists of two parts (blue arrows in Fig. 2): (1) historical air quality (PM2.5), latitude and longitude; historical meteorological data (weather, temperature, pressure, humidity, wind speed, wind direction) of the target site and nearby sites. ; (2) Weather forecast data (weather, wind direction, wind level, up and down temperature) of the target site. The output of WLSTME model is the PM2.5 forecast value of the target site the next day.

#### MLP for generating the weighted PM2.5

MLP can theoretically approximate any Borel measurable function with arbitrary precision^25^. We constructed a three-layer MLP spatial correlation processor to generate weighted PM2.5 series data for each neighbor site. Neighbor sites were defined as the K nearest surrounding sites to the central site. Since pollutants are transported among areas based on wind, air pollution of the central sites are spatially correlated with that of neighbor sites. However, the distribution of monitoring stations is not even. Consequently, the distance between neighbor sites and the central site is different for different central sites. For example, the density of stations in the south area is much sparser than Beijing, as Fig. 1 shows. Thus, for central sites in the south area, the selected neighbor sites were more distant, and the spatial correlation was lower than that for sites in Beijing. Based on the above consideration, the geographical distance of the selected neighbor sites should be considered in the model.

**Figure 3:**
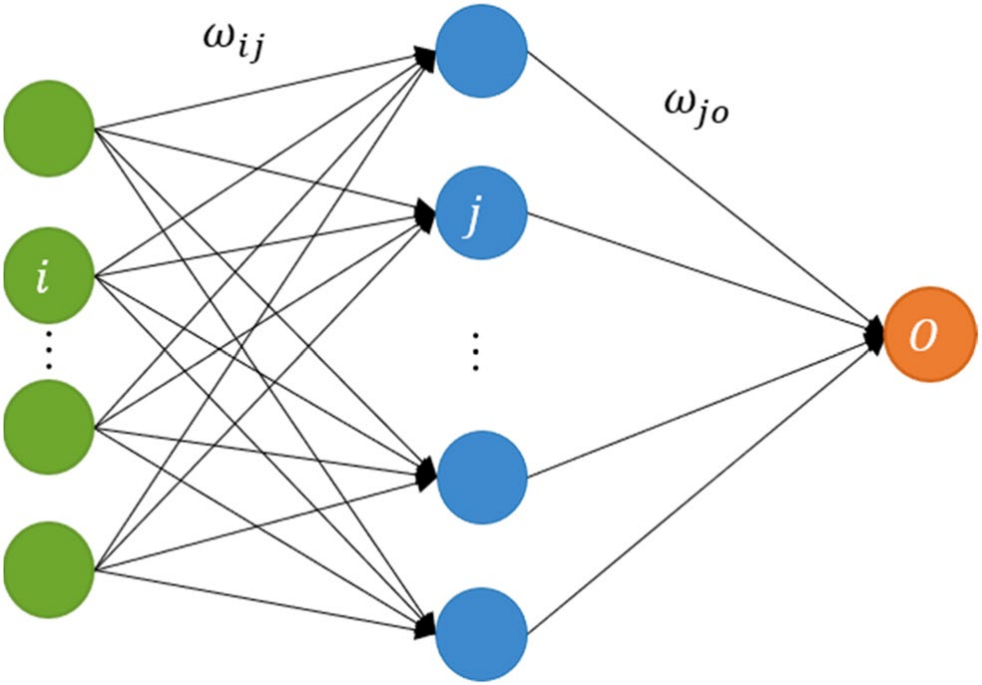
The structure of the three-layers MLP model.

The three-layer MLP integrates the distance and wind of neighbor sites with its PM2.5 to generate weighted PM2.5 data for each neighbor site j of central site i. Figure 3 shows the structure of MLP. Given ${PM2.5}_{ jt}$ and ${v}_{jt}$ represent the PM2.5 concentration and wind speed of the $jth$ neighbor at time *t,* respectively; ${d}_{ij}$ represents the distance between the central site $i$ *a*nd its $\mathrm{jth}$ neighbor site; ${\uptheta }_{ijt}$ represents the angle between the wind direction of $\mathrm{jth}$ neighbor site at time $t$ and the edge between $i$ and $j$. ${H}_{1},\dots ,{H}_{n}$ are neurons of the hidden layer, and ${WPM2.5}_{jt}$ is the weighted PM2.5 concentration, which is calculated by the Eqs. (5) and (6).

5 $${H}_{s}={g(\omega }_{1s}^{1}{PM2.5}_{jt}+{\omega }_{2s}^{1}{d}_{ij}+{\omega }_{3s}^{1}{v}_{jt}+{\omega }_{4s}^{1}{\theta }_{ijt}$$

6 $${WPM2.5}_{ jt}={\sum }_{s=1}^{n}{\omega }_{s}^{2}{H}_{s}$$

where $g$ is the activation function used for the nonlinear transformation of inputs. $\omega$ is the weight between the neuron of previous layer and the next layer.

#### LSTM for extracting spatial–temporal feature

**Figure 4:**
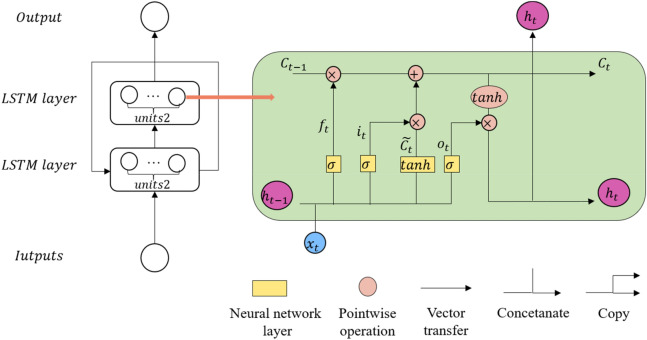
The illustration of two-layers LSTM model. ${x}_{t}$ and ${h}_{t}$ are the inputs and outputs at time ***t*** respectively.

LSTM is a special recurrent neural network, with its recurrent neuron simultaneously captures long and short dependencies in time series data. LSTM has been used in many fields, such as financial market predictions^26^, epileptic seizures^27^, and reservoir operation^28^. All of the LSTM models used in these fields exhibited better performance than many other machine learning methods. The LSTM model used in our model was a two-layer stateful LSTM, which used the state of the current batch of LSTM samples as the initial state of the next batch of samples. It is more suitable for processing long-term time series data than the other models. The structure of the recurrent memory cell of the LSTM model is shown in Fig. 4.

The bottom layer of the proposed LSTM FRAME corresponds to the input layer. The middle core layer comprises two LSTM layers, and the output layer follows. Each neuron of the LSTM layer has an architecture similar to that in the right part of Fig. 4. Three key gates, namely, forget gate ($${f}_{t}$$), input gate ($${i}_{t})$$, and output gate ($${o}_{t})$$, of LSTM are designed to control the memory of new information and to forget old information. The values of the three gates are updated with time respectively by Eqs. (7), (8), and (9).

7 $$f_{t} = \sigma \left( {W_{f} \cdot \left[ {h_{t - 1} ,x_{t} } \right] + b_{f} } \right),\;\;i_{t} = \sigma \left( {W_{i} \cdot \left[ {h_{t - 1} ,x_{t} } \right] + b_{i} } \right),$$

8 $$\widetilde{{C_{t} }} = tanh\left( {W_{C} \cdot \left[ {h_{t - 1} ,x_{t} } \right] + b_{C} } \right),\;\;C_{t} = f_{t} \cdot C_{t - 1} + i_{t} \cdot \widetilde{{C_{t} }}$$

9 $$o_{t} = { }\sigma \left( {W_{o} \cdot \left[ {h_{t - 1} ,x_{t} } \right] + b_{o} } \right),\;\;h_{t} = o_{t} \cdot tanh\left( {C_{t} } \right)$$

where ${x}_{t}$ and ${h}_{t}$ are the inputs and outputs of time $t$, respectively; $\sigma$ and hyperbolic tangent are widely used activation functions; $W$ and $b$ are the weight matrix and bias vector, respectively.

In detail, LSTM is designed to extract spatiotemporal features from the pollution data of central site and neighbor sites to make a prediction. The weighted PM2.5 series data of neighbor sites and PM2.5 concentration observation data of the central site were merged as a 2D matrix, with each column represented the historical PM2.5 concentration of the central site or weighted PM2.5 concentration of a neighbor site. The size was $\mathrm{r}\times (\mathrm{K}+1)$, where r was time lag, and K was the number of selected neighbor sites. We placed this matrix into a two-layer LSTM model so that their spatial and temporal dependence and synthetic action could be considered simultaneously. The output of the LSTM model is called pre-prediction.

#### MLP weather forecasts optimizer

Finally, auxiliary variables were introduced to the WLSTME model to promote prediction accuracy. The auxiliary variables considered in this study included meteorological data (temperature, wind speed, dew point temperature, mean sea level pressure and total column water vapor), time stamp data (day of week and month of the year), and latitude of the central site at time *T*. We integrated the auxiliary variables with the spatiotemporal features extracted by LSTM and input them into MLP to output the prediction of the next day’s PM2.5 concentration of the central site. The structure of MLP was the same as Fig. 3, however, the input and output were substituted by the spatiotemporal features and PM2.5 concentration prediction, respectively.

### Evaluation methods

We predict real-values of PM2.5 concentration for the next day. Three criteria, namely, mean absolute error (MAE), root mean square error (RMSE), and total accuracy index (p), were used in the experiments to evaluate the effectiveness of our model. Their definitions are given in Eqs. (10), (11) and (12):

10 $$MAE=\frac{1}{n}\sum_{\begin{array}{c}\\ \\ i=1\end{array}}^{n}\left|{y}_{i}-{y}_{i}^{*}\right|$$

11 $$RMSE=\sqrt{\frac{1}{n}\sum_{i=1}^{n}{({y}_{i}-{y}_{i}^{*})}^{2}}$$

12 $$p=1-\frac{\sum_{i=1}^{n}\left|{y}_{i}-{y}_{i}^{*}\right|}{\sum_{i=1}^{n}{y}_{i}}$$

where $n$ is the number of samples, ${y}_{i}$ is the observation of the $\mathrm{i}$th sample, and ${y}_{i}^{*}$ is the $\mathrm{i}$th forecasting value. In addition, we employed the spatial anomaly correlation (ACC) and the temporal correlation coefficient (TCC) as the evaluation metrics, which are defined as in the following equations:

13 $$\stackrel{-}{{y}_{i}}=\frac{1}{N}{\sum }_{j=1}^{N}{y}_{ij}, \quad \stackrel{-}{{y}_{i}^{*}}=\frac{1}{N}{\sum }_{j=1}^{N}{y}_{ij}^{*}$$

14 $${\Delta y}_{ij}={y}_{ij}-\stackrel{-}{{y}_{i}}, \quad {\Delta y}_{ij}^{*}={y}_{ij}^{*}-\stackrel{-}{{y}_{i}^{*}}$$

15 $$ACC=\frac{1}{N}{\sum }_{j=1}^{N}\frac{{\sum }_{i=1}^{M}({\Delta y}_{ij}-\stackrel{-}{{\Delta y}_{j}})({\Delta y}_{ij}^{*}-\stackrel{-}{{\Delta y}_{j}^{*}})}{\sqrt{{\sum }_{i=1}^{M}{({\Delta y}_{ij}-\stackrel{-}{{\Delta y}_{j}})}^{2}{\sum }_{i=1}^{M}{({\Delta y}_{ij}^{*}-\stackrel{-}{{\Delta y}_{j}^{*}})}^{2}}}$$

16 $$TCC=\frac{1}{M}{\sum }_{i=1}^{M}\frac{{\sum }_{j=1}^{N}({y}_{ij}-\stackrel{-}{{y}_{i}})({y}_{ij}^{*}-\stackrel{-}{{y}_{i}^{*}})}{\sqrt{{\sum }_{j=1}^{N}{({y}_{ij}-\stackrel{-}{{y}_{i}})}^{2}{\sum }_{j=1}^{N}{({y}_{ij}^{*}-\stackrel{-}{{y}_{i}^{*}})}^{2}}}$$

where ${y}_{ij}$ and ${y}_{ij}^{*}$ represent the observed and predicted value of station $i$ in day $j$, respectively. $M$ and $N$ represent the count of stations and days of the test, respectively.

## Results and discussion

The experimental data include the daily air pollutant observations, meteorological factor observations, and weather forecasts of the 90 stations in the BTH area from 1 January 2015 to 31 December 2017. A total of 98,640 samples were collected for this area (more details in “Study Area”). To avoid future values used to predict the past, we divide the data set according to the time sequence. The data are broken down by year rather than by month, as many studies^29,30^ show apparent seasonal differences in PM2.5 concentrations. Therefore, we divide the observed data into three datasets of 2015, 2016, and 2017, respectively, according to the time sequence. The datasets of 2015 and 2016 are used for model training and validation, respectively. The dataset of 2017 used to test and to evaluate the performance of the proposed model. Based on the three datasets, we completed the model training and daily PM2.5 prediction and evaluation and comparative experiments. Section “Parameter settings of Model training” introduces parameter settings of model training, and “Parameter settings of Model training” and “Prediction and evaluation and comparative experiment” presents the experimental results and discusses the advantage of the proposed methods.

### Parameter settings of model training

Several parameters, including time lags ($r$), number of considered neighbor sites ($\mathrm{s}$), number of neurons in the hidden layer in the first MLP, LSTM, and the final MLP (marked by $\mathrm{units }1, 2,\mathrm{ and }3$ respectively), and batch size of training, were preset before building our WLSTME model. We employed partial autocorrelations to measure the time dependency of 90 stations. When time $lag=10$*.*, almost 30 stations showed a correlation larger than 0.1, indicating the existence of non-negligible correlation when time $lag=10$*.* We selected 7, 8, 9, and 10 as candidates of time lags $r$. Figure 1 shows that most stations gathered together in a group of 4 to 10, excluding Beijing, which has 35 stations inside. Thus, we selected 5, 10, 15, and 20 as candidates of the number of neighbor stations $s$. $\mathrm{Units }1$, $2$ and $3$ were selected in 10, 20, 30, and 40, and the chosen batch size was in $(1, \dots , 7)\times 90$*.*

The best parameters were determined through a trial-and-error method based on the training and validation sets. The loss function was set to mean square error function, and the optimizer was set to "RMSprop", which is widely used in the training of neural network. Finally, the best parameters were determined, where $r=10, s=10, unit1=10,unit2=10,unit3=20$ and $\mathrm{batch }size=7\times 90$.

### Prediction and evaluation and comparative experiment

Firstly, the model training and daily PM2.5 prediction and evaluation were completed based on the 3 datasets. To further verify the performance of the proposed model, we conducted comparative experiments between our model and other models that consider spatiotemporal dependency, such as GWR and LSTME. LSTME has the same architecture as our model, except for the first MLP network, which means that the historical PM2.5 data of k neighbor sites at past $\mathrm{r}$ days were directly merged with the data of the central station and formed the inputs of the LSTM layers. In addition, the performance of STSVR, a spatiotemporal model with wind considered, was also compared; STSVR was proposed by Yang et al.^21^.

Table 2 shows the performance of GWR, LSTME, STSVR, and WLSTME models in the test set. The models that consider wind in spatiotemporal dependency (STSVR and WLSTME) exhibited better forecasting performance than GWR and LSTME and had lower $\mathrm{RMSE}$, lower $\mathrm{MAE}$, and higher $p$. These results indicate that the influence of wind on the spatiotemporal correlations among stations is significant, and considering wind can considerably promote the accuracy of model predictions. In comparison with STSVR, WLSTME has lower $\mathrm{RMSE}$, lower $MAE$, and higher $p$. Additionally, the ACC and TCC of WLSTME model was higher than that of STSVR, indicating that the MLP network can better fit the correlation between wind and spatiotemporal dependency than the Gaussian form.

**Table 2:** The prediction performance of GWR, LSTME, STSVR and WLSTME models on the test set.

	$\mathrm{RMSE}$	$\mathrm{MAE}$	$\mathrm{p}$	ACC	TCC
GWR	49.34	35.84	0.43	0.9059	0.9658
LSTME	51.81	36.58	0.42	0.8971	0.9668
STSVR	42.60	27.80	0.56	0.8983	0.9867
WLSTME	**40.67**	**26.10**	**0.59**	0.9524	0.9930

Bold means best achieved results.

In addition, in order to test the over-fitting degree of these models, we split the whole dataset (including the data of 2015, 2016, and 2017) into five subsets and conducted cross-validation experiments. The cross-validation performances of the four models are presented in Table 3. It can be seen that the proposed WLSTME model still performed the highest accuracy. The RMSE, MAE of all models were not much smaller than that of the test set; thus, all of these models did not show obvious over-fitting problems.

**Table 3:** The cross-validation performance of GWR, LSTME, STSVR and WLSTME models.

	$\mathrm{RMSE}$	$\mathrm{MAE}$	$\mathrm{p}$
GWR	50.73	34.51	0.52
LSTME	52.62	35.89	0.50
STSVR	48.10	30.77	0.57
WLSTME	**43.14**	**29.15**	**0.59**

Bold means best achieved results.

#### Temporal heterogeneity of predictions

To compare the temporal heterogeneity of the various models, the test set of 2017 was divided into four seasons according to meteorological standards: spring (March to May), summer (June to August), autumn (September to November), and winter (January, February, and December). To compare the temporal heterogeneity of the prediction performance of various models, we obtained their *RMSE*, *MAE,* and p results in different seasons separately.

**Figure 5:**
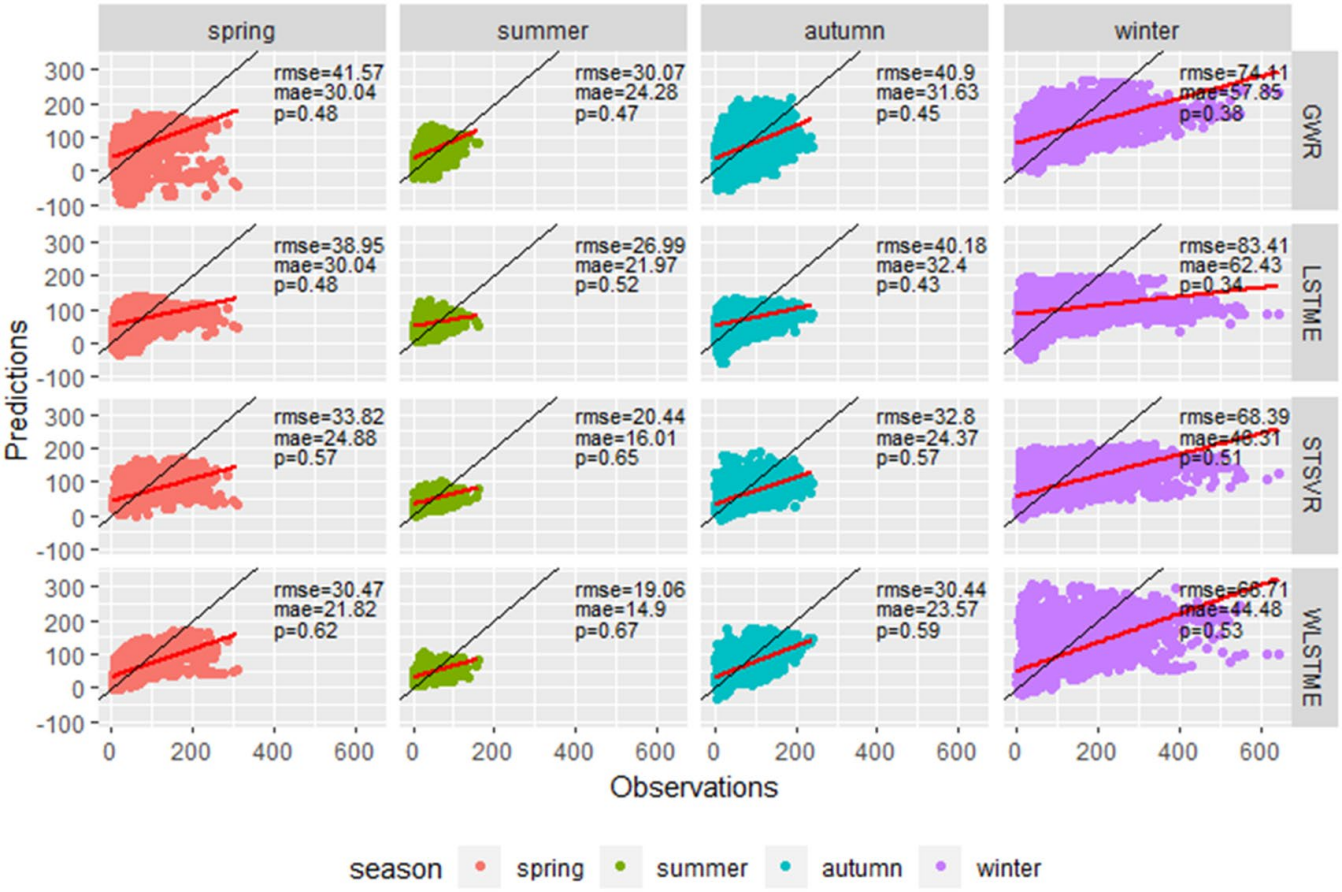
The performance of GWR, LSTME, STSVR and WLSTME models under four seasons of 2017. The black lines mean $y=x$, and the red lines are separate fitting lines between predictions of four models and observations in different seasons.

**Figure 6:**
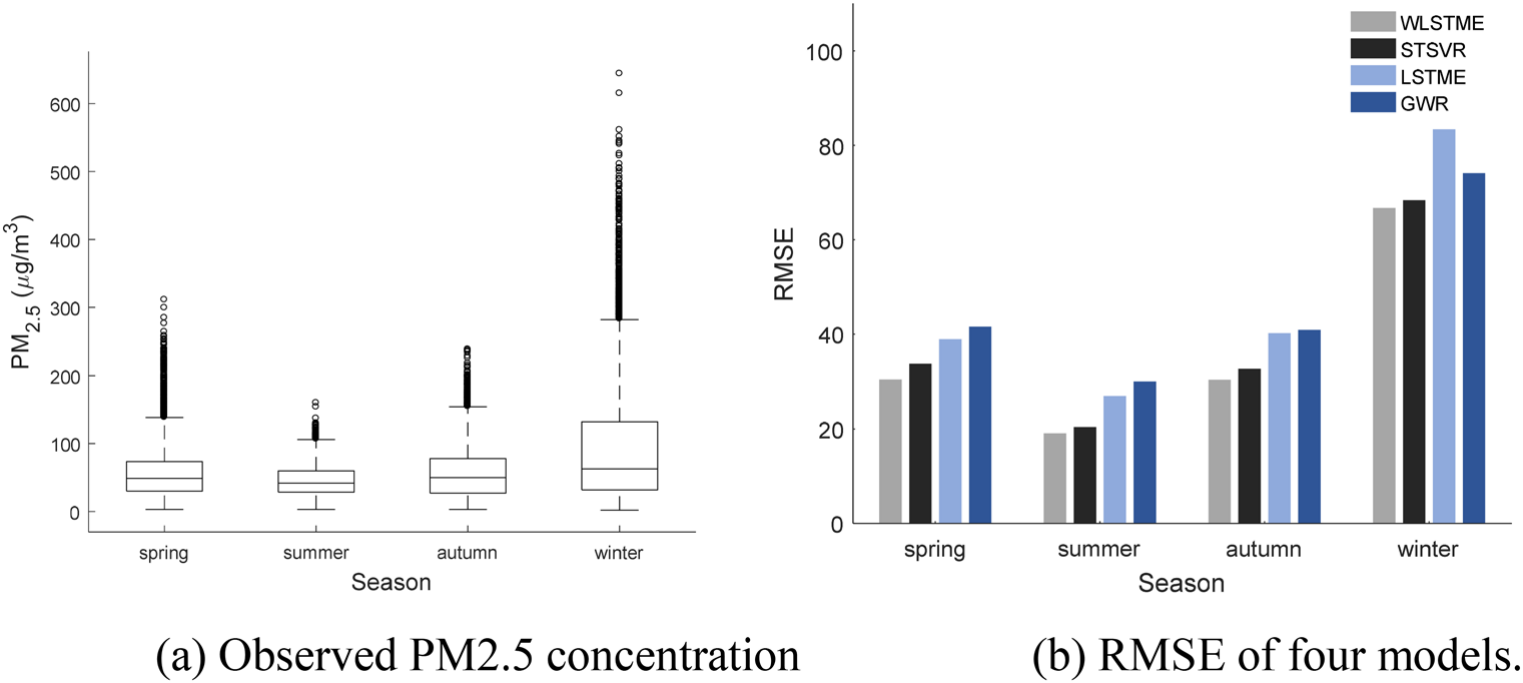
Seasonal comparison of four models.

Figure 5 shows the performance of GWR, LSTME, STSVR, and WLSTME in different seasons. The different colored dots represent observations and corresponding predictions in different seasons, and the black lines mean $y=x$. Figure 6 shows the seasonal comparison of both observed PM2.5 concentration and $RMSE$ of four models.

According to both Figs. 5 and 6a, summer is the least polluted (the lowest mean) and most stable season (the shortest interval of distribution). Some relatively small peak values occurred in spring and autumn, and extremely high values happened in winter. Considering that peak values cause the prediction more difficult, the prediction performance of four models were the best in summer, followed by spring and autumn, and winter is the worst season. Under four seasons, the proposed WLSTME model outperformed all other models with slight difference in its advantage. In summer, the $RMSE$ of WLSTME was 1.38 lower than STSVR, which was the smallest promotion among four seasons. In spring and autumn, the promotion of WLSTME compared with STSVR was more significant: $RMSE$ was reduced by 3.35 and 2.36, respectively. In spring, p increased by 0.05, which illustrated the superiority of WLSTME model in predicting peak values. Winter was comparatively difficult to predict because of the extreme peak values, which may be due to fuel burning and fireworks. Without any economical and policy information, the effectiveness of all models decreased. Nevertheless, WLSTME still achieved the highest prediction accuracy. In short, WLSTME achieved good performance in all seasons and exhibited remarkable prediction accuracy of peak values.

#### Spatial heterogeneity of predictions

**Figure 7:**
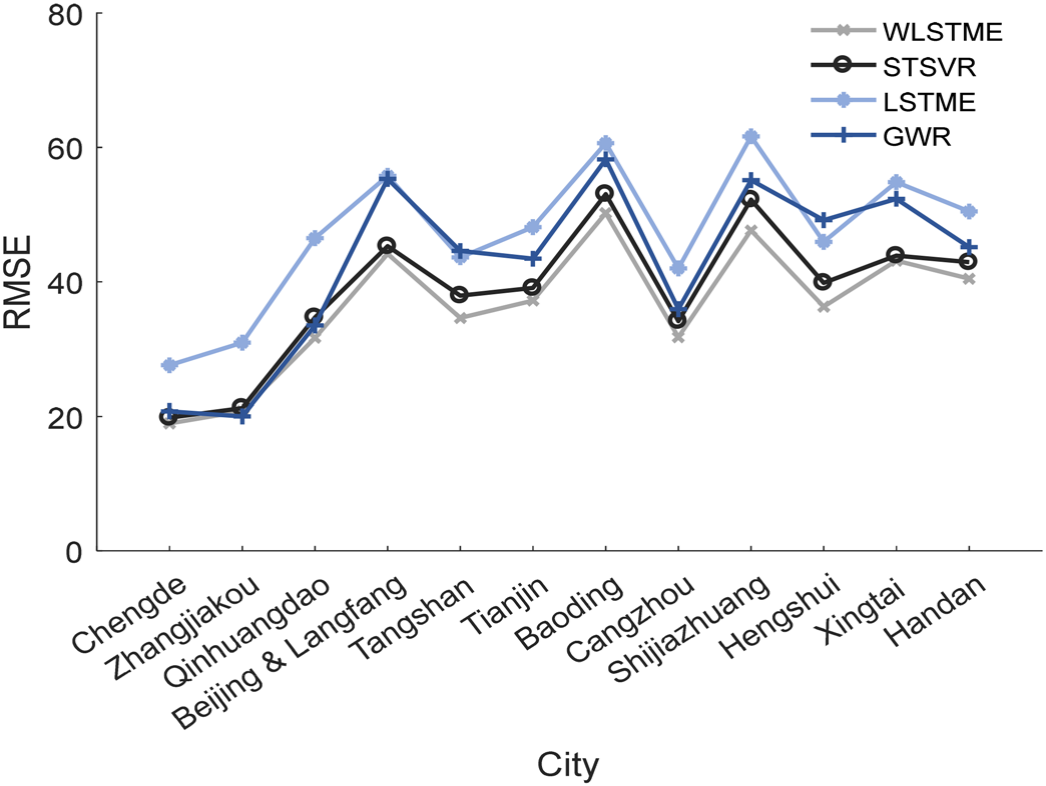
Comparison results of the RMSE of four models in different cities.

Considering that a low latitude equates to a high average daily PM2.5, according to Fig. 1, we divided the 90 stations into 12 groups based on their city to investigate the spatial heterogeneity of predictions. It is noted that due to the monitoring sites of Langfang are near to the sites of Beijing; these sites were merged together into one group. Figure 7 shows the comparison results of the RMSE of the four models in different groups. The cities are arranged in the descending order.

The RMSE values presented an increasing trend, demonstrating that as the latitude decreased, the difficulty of prediction increased. WLSTME performed the best in all cities, and a relatively large gap between the RMSE of WLSTME and other models was observed, especially in cities with a lower density of stations, such as Tangshan, Shijiazhuang, Hengshui, and Handan. However, in the cities with lots of sites, such as Beijing and Tianjin, the superiority of WLSTME is relatively lower. These results indicate that WLSTME can better simulate spatiotemporal dependency through using MLP to combine wind, distance, and PM2.5 concentration in stations sparsely distributed areas.

## Conclusion

In this study, we developed a WLSTME model to predict the daily average PM2.5 concentration of a specific station with the uneven distribution of monitoring sites were considered. First, MLP was used to combine historical wind speed and wind direction with corresponding days’ PM2.5 data of neighbor sites and generate weighted PM2.5. Second, the weighted PM2.5 of the neighbor sites in the past 10 days were merged with the historical PM2.5 data of the central station, and input into LSTM layer to address the spatial and temporal dependence simultaneously and extract spatiotemporal features. Finally, another MLP was used to conduct bias adjustment by integrating the spatiotemporal features with the central site’s meteorological data and time stamp data.

Comparative experiments were conducted on the data of 2017 in the BTH area. *RMSE*, *MAE*, and *p* were used to quantify the forecasting performance and calculated in all subsets, which were grouped by season or city. All results showed that in each season and region, WLSTME performed higher predictive accuracy and reliability than STSVR, LSTME, and GWR, especially in spring and autumn. The main reason for this superiority is that WLSTME not only addresses spatial and temporal dependencies simultaneously but also exhibited an exceptional capability to simulate the impact of wind and geographical distance on such dependencies.

In the future, the focus should be put on the prediction of the sudden increase in PM2.5, especially in winter when all models performed poorly. The inherent laws of winter could be different from those of other seasons, and a specific model can enhance accuracy. Additionally, other humanistic and economic factors, such as the influence of government policies and the number of factories in the area, should be introduced. Finally, more sophisticated methods for considering the density of sites can be investigated.
